# Clinical outcomes and aesthetic results of reverse sequence endoscopic versus traditional bilateral nipple-sparing mastectomy with immediate implant-based breast reconstruction-an analysis of initial 116 patients from single institution

**DOI:** 10.3389/fonc.2025.1496592

**Published:** 2025-03-10

**Authors:** Qing Zhang, Faqing Liang, Juan Li, Yanyan Xie, Yu Feng, Mengxue Qiu, Jiao Zhou, Huanzuo Yang, Qing Lv, Zheng-gui Du

**Affiliations:** ^1^ Department of General Surgery, West China Hospital of Sichuan University, Chengdu, China; ^2^ Breast Center, West China Hospital of Sichuan University, Chengdu, China; ^3^ Department of Breast Surgery, Sichuan Provincial People’s Hospital, University of Electronic Science and Technology of China, Chengdu, China; ^4^ Department of General Surgery, The Fourth People’s Hospital of Sichuan Province, Chengdu, China

**Keywords:** breast cancer, reverse-sequence, endoscopic surgery, bilatral breast reconstruction, traditional surgery

## Abstract

**Background:**

Endoscopic or robotic surgeries can minimize and hide the scars compared to conventional breast reconstruction but are considered unsuitable for bilateral procedures due to the extended operation time. This study explored a novel time-shortening endoscopic technique, namely reverse-sequence endoscopic nipple-sparing mastectomy (R-E-NSM) with bilateral implant-based breast reconstruction (BIBR), and compared it with conventional open surgery in clinical and cosmetic outcomes.

**Methods:**

We retrospectively analyzed patients who underwent BIBR in the West China Hospital from January 2017 to June 2022. Patient characteristics, operation time, postoperative complications, breast satisfaction, and Scar-Q scores were compared between endoscopic and conventional open groups.

**Results:**

Among 116 patients, 76 underwent R-E-NSM with BIBRs (R-E-BIBR group), and 40 underwent conventional open BIBRs (C-O-BIBR group). The demographics and clinical data were similar primarily (P > 0.05). Compared with the C-O-BIBR group, the R-E-BIBR group had lower rates of total (32.5% versus 6.6%, P < 0.001), major (13.8% versus 2.0%, P < 0.001) and minor (23.8% versus 3.9%, P < 0.001) complications. The operation time between the two groups is not statistically significant (290.2 ± 95.2 mins versus 271.9 ± 95.3 mins, P = 0.327). The Harris scale scored breast satisfaction, and the excellent rate of the C-O-BIBR group was 32.5% while the R-E-BIBR group was 58.0% (P < 0.001). The mean Scar-Q scores were 35.17± 9.6 in the C-O-BIBR group and 81.32 ± 12.3 in the R-E-BIBR group, respectively (P < 0.001).

**Conclusion:**

The innovative R-E-NSM with implant-based breast reconstruction makes up for the long operation time of previous endoscopic surgeries and has significant advantages in reducing complication rates and improving the cosmetic results of the postoperative breasts.

**Level of Evidence:**

Level III, Retrospective study.

## Introduction

Bilateral breast reconstruction is of great importance for patients with bilateral breast cancer, who are under tremendous psychological pressure due to the physical defects caused by the loss of both breasts (1). Many patients with unilateral breast cancer undergo unilateral breast reconstruction also want to conduct contralateral prophylactic mastectomy and reconstruction at the same time because they are worried about the development of contralateral breast cancer (2–4). Moreover, patients with BRCA mutations, severe atypical dysplasia hyperplasia of both breasts or a strong family history often require bilateral prophylactic mastectomy and reconstruction (4, 5). Therefore, the clinical importance of bilateral breast reconstruction is self-evident because it is suitable for a vast population (6–8). The common methods of bilateral breast reconstruction include bilateral autologous breast reconstruction (ABR), unilateral ABR with contralateral implant-based breast reconstruction (IBR), and bilateral IBR (BIBR) (9, 10). Some studies have shown that ABR is superior to IBR in unilateral breast reconstruction (11). However, BIBR is less invasive than bilateral ABR and can obtain better cosmetic results, so it has become the first choice for bilateral breast reconstruction (12).

Although BIBR can significantly improve the psychosocial health of these patients, the symmetry problem and obvious scars, especially the scar of the radial incision of the lateral breast or nipple, have also become deep distress for patients who choose this surgery (12–14). Although the inframammary fold (IMF) incision is concealed and hidden in the standing position because of the breast ptosis, the exposure of symmetrical scars in both breasts in the supine position is unacceptable (15, 16). Besides, the IMF incision is only suitable for small to medium-sized, slightly ptotic breasts (17). For breasts without ptosis, the scar cannot be hidden; For larger and/or severe ptosis breasts, the superior part of the gland cannot be completely removed, so the indications for this surgery through the IMF incision are also limited (18). Thus, it is of great importance to find the perfect incision for IBR after bilateral mastectomy, especially when it can be applied to a broader population.

Bilateral breast augmentation via axillary incision provides us with a good idea. However, removing the gland through bilateral axillary incisions is challenging with conventional open surgery (19). Endoscopic and robotic surgery can transfer and shorten the surgical incision to the axilla (20, 21). Still, conventional endoscopic or robotic surgery is difficult to be widely used because of the multiple incisions, multiple instruments, trauma, and long operation time, so it is only conducted in a few medical centers (22). Let alone endoscopic or robotic bilateral breast reconstructions, which are rarely reported and just have small populations. However, three years ago, we explored a reverse-sequence endoscopic nipple-sparing mastectomy (R-E-NSM) with IBR, which could reduce the operation time significantly, further reduce the incidence of surgical complications without special equipment requirements, and obtain better cosmetic results and higher patient satisfaction (23–26). We even conducted R-E-NSM with DIBR in the 24-hour surgery center and pioneered R-E-NSM and immediate breast reconstruction with reverse-sequence endoscopic latissimus dorsi muscle harvesting (24–31). As a result, R-E-NSM with BIBR has been established as a standard surgical procedure at our center. In this study, we will compare the safety, postoperative cosmetic results, and operation time of the R-E-NSM with BIBR and conventional open BIBR procedures.

## Patients and method

### Study population

One hundred and sixteen consecutive patients who underwent bilateral nipple-sparing mastectomy (NSM) with BIBR at our hospital from January 2017 to June 2022 were retrospectively selected. For patients who underwent bilateral mastectomy, the breast shape after open reconstruction and endoscopic reconstruction was shown by the previous postoperative prognosis diagram, and the possible differences in complications and oncologic safety were introduced. Finally, the choice of reconstruction method was decided by the patient. All data were prospectively collected, and databases were maintained by professionals. Due to this article is a retrospective study, it does not require IRB approval. Based on the surgical methods, the patients were divided into the reverse-sequence endoscopic group (R-E-BIBR group, N=76) and the conventional open group (C-O-BIBR group, N=40).

**Figure 1:**
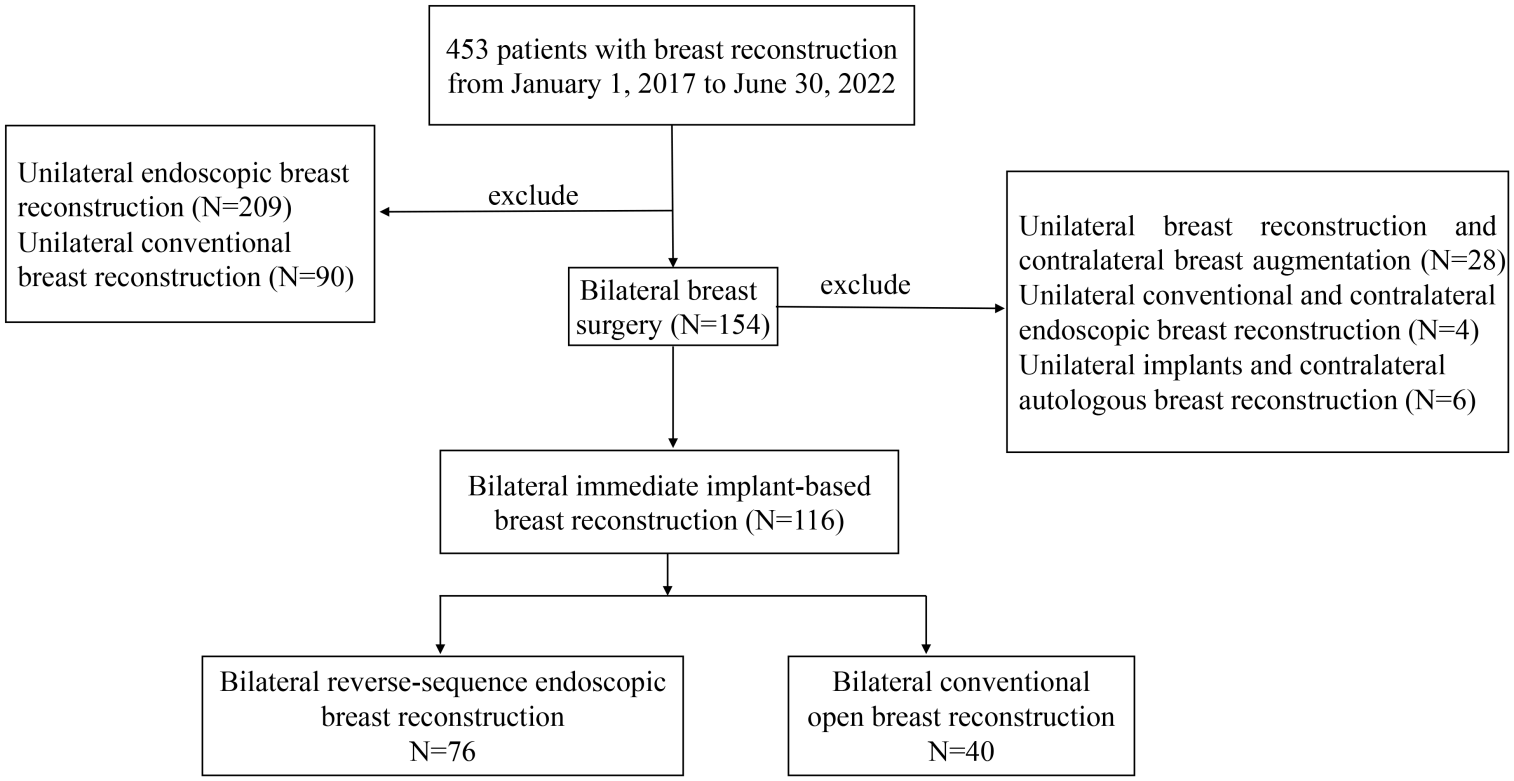
Flow chart of date selection.

Inclusion criteria were as follows: female patients ≥ 18 years; tumor size ≤ 5 cm on preoperative imaging; no clinical and radiological evidence of skin, nipple-areolar complex (NAC), and chest wall invasion before and after neoadjuvant chemotherapy; no evidence of multiple lymph node metastasis (cN0 and cN1); preoperative pathology confirmed bilateral or unilateral breast cancer or bilateral breast atypical hyperplasia, or BRCA1/2 mutations with a strong family history. Patients with Paget disease, recurrent breast cancer, or a history of previous thoracic radiation therapy were not eligible. Additionally, patients were not eligible if they were pregnant, had a high American Society of Anesthesiology score (>2), had uncontrolled diabetes mellitus, were prior or current heavy smokers (>20 cigarettes/day), had previous surgery in ipsilateral breast, underwent unilateral reconstruction, unilateral implant breast reconstruction and contralateral breast augmentation, unilateral conventional and contralateral endoscopic breast reconstruction, unilateral implants and contralateral autologous breast reconstruction or delayed procedures (**Figure 1**).

### Surgical methods

In the C-O-BIBR group, after sentinel lymph node biopsy (SLNB) or axillary lymph node dissection (ALND), a lateral radial incision (6-12 cm), tumor surface incision (6-12 cm), periareolar arc incision (3-6 cm), or IMF incision (10-15 cm) was made, and NSM was performed. Then, the subpectoral layer, if needed (subpectoral or dual-plane breast reconstruction), was dissociated by an electric scalpel for forming the implant pocket, and the predetermined-size prosthesis or tissue expander was placed to complete the reconstruction, which was detailed in the references (13).

**Figure 2:**
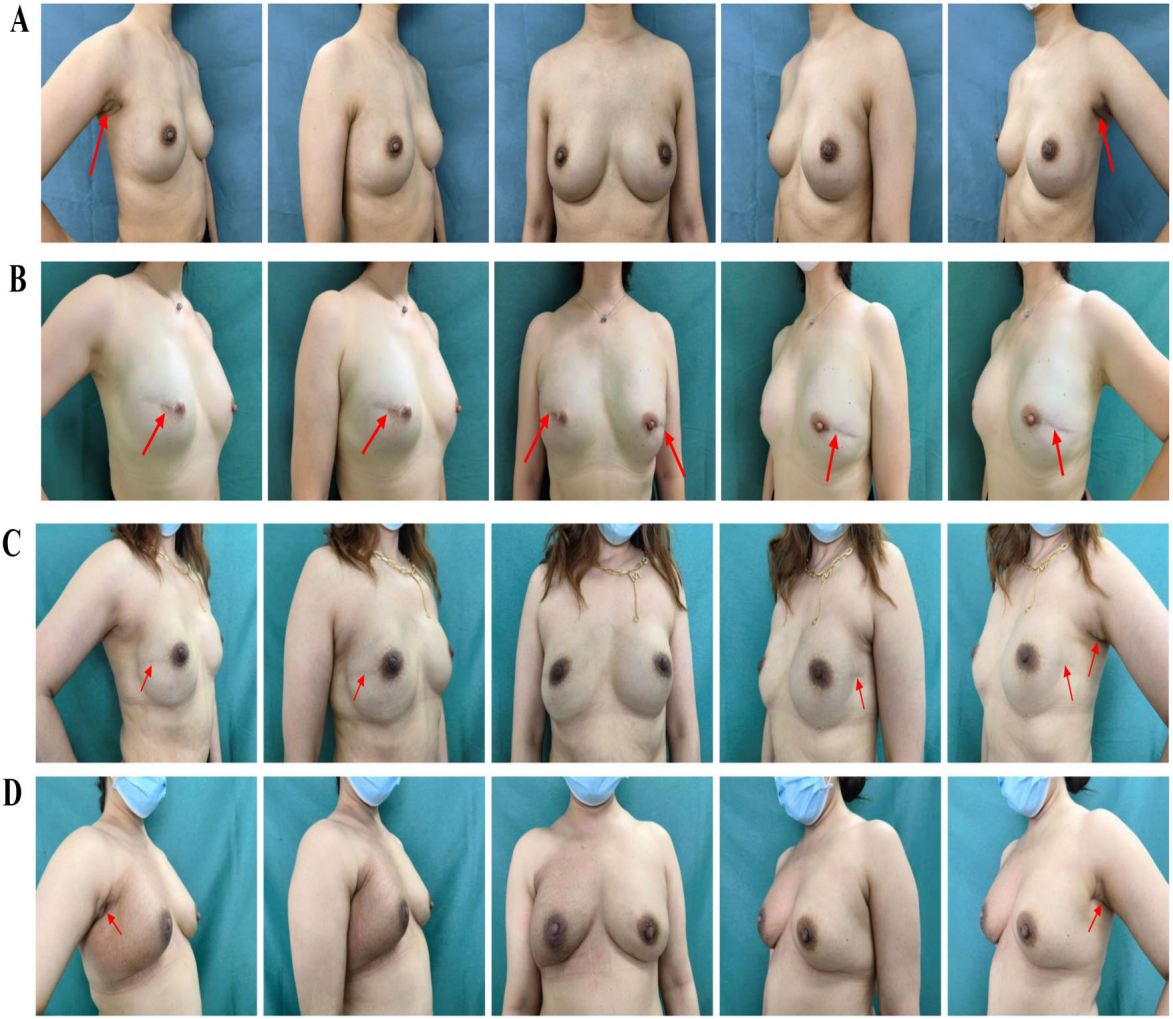
Postoperative comparison between the two group. **(A)** is a 42-year-old patient with atypical hyperplasia in both breasts with BRCA1 mutation who underwent bilateral reverse-sequence endoscopic mastectomy and immediate implant-based reconstruction, and this is her photo 1 year after surgery. The red arrow points to the scar position. **(B)** is a 37-year-old patient with bilateral triple-negative breast cancer who underwent bilateral conventional open mastectomy and immediate implant-based reconstruction, and this is her photo 3 years after surgery. The red arrow points to the scar position. **(C)** is a 31-year-old patient who was diagnosed with left breast carcinoma (T1N0M0) and underwent bilateral conventional open mastectomy and immediate implant-based reconstruction, this is her photo 3 years after surgery. The red arrow points to the scar position. **(D)** is a 35-year-old patient who was diagnosed with right breast carcinoma (T1N1M0) underwent bilateral reverse-sequence endoscopic mastectomy and immediate implant-based reconstruction, and underwent radiotherapy after surgery. This is her photo 1 year after radiotherapy. The red arrow points to the scar position.

The R-E-NSM procedure will make a 5-7cm incision at the sub-axillary fold and first perform axillary management. After establishing the working space, the procedure was conducted in the reverse sequence (“from subpectoral to retromammary space and then to subcutaneous plane”) through the less visible axillary incision. If the surgeon performs pre-pectoral reconstruction, there is no need to dissect the layer of the posterior pectoral fascia. In addition, we created a 2 mm accessory incision on the outer-upper edge of the areola (named “HUAXI Hole 1”), which could be used to easily dissociate the glands in the lower-inner quadrant that was difficult to dissect through the axilla approach. Finally, the gland was removed from the axillary incision completely, and the implant (single or combined with mesh) was inserted into the subpectoral or pre-pectoral pocket. The surgical procedures have been detailed in our previous articles, and the surgical instruments are simple and available in almost all hospitals, as shown in **Figure 2** (23, 25).

### Data collection

The postoperative complications, patient-reported outcomes, operation time, and anesthesia time were compared between the R-E-BIBR and C-O-BIBR groups. The Clavien–Dindo classification (CDC) was developed to define and grade postoperative events (32). Postoperative complications included major and minor complications. Major complications: according to the CDC classification criteria, grade III or IV was considered as major complications, which required surgical intervention or reoperation, such as implant loss caused by infection. Minor complications: according to the CDC classification criteria, grade I or II was considered as minor complications, that is, complications that can be controlled only by observation or oral drugs, such as seroma and infection that can be controlled by oral antibiotics. The breast satisfaction between the two groups was compared using the Harris and Ueda scale at the last follow-up. Patients performed the Harris score, and surgeons performed the Ueda score (33, 34). The Harris score: It enables patients to conduct a cosmetic evaluation of the reconstructed breast in terms of volume, position, and effect. This scale is divided into four grades: excellent, good, fair, and poor. The Ueda score: It is an evaluation method for the cosmetic outcome after breast reconstruction by professionals such as doctors. The score results are: excellent (≥9 points); good (7-8 points); acceptable (5-6 points); and poor (≤4 points). The surgical incision scar was compared using the Scar-Q appearance scale (35). The Scar-Q score is a validated comprehensive patient-reported outcome measure for evaluating scars. It comprises three dimensions: scar appearance, scar symptoms, and psychosocial impact, with a score range of 0 to 100, where a higher score indicates a better outcome. Follow-up timeline, postoperative 1 month, 3 months, 6 months, 12 months, 24 months, the end of follow-up time. Scar-Q scores were completed by patients at the last follow-up and collected by trained medical assistants not involved in the surgery.

### Statistical analysis

Categorical data are presented as numbers and percentages, compared by chi-square test, and continuous data are presented as mean ± standard deviation (x ± s), compared by independent sample Student’s t-tests. While ranked data were compared by Wilcoxon rank sum test. And for cases of baseline mismatch, Univariate and multivariate logistics regression analyses were performed for adjustment. In the multivariate logistic regression of complications, surgical complication occurrence was regarded as the dependent variable, and all other baseline data were considered independent variables for univariate logistic regression. Variables with P < 0.1 among them were incorporated into the multivariate logistic regression. In the multivariate logistic regression of satisfaction, satisfaction grouping was taken as the dependent variable, and all other baseline data were regarded as independent variables for univariate logistic regression. Variables with P < 0.1 among them were included in the multivariate logistic regression. Linear regression was used to predict the change in operation time and compared by Student’s t-tests. Prognostic indicators were analyzed by Kaplan-Meier. Statistical significance was set at two-sided P < 0.05. SPSS v26.0 (IBM SPSS Inc., Armonk, NY, USA) was used for statistical analysis. We used GraphPad Prism 8 and Adobe Photoshop 2022 for statistical mapping.

## Results

### Demographic and clinical characteristics

A total of 116 consecutive patients who underwent BIBR from January 2017 to June 2022 were analyzed. The R-E-BIBR group included 76 patients, while the C-O-BIBR group included 40 patients. The median follow-up time was 22 months in the R-E-BIBR group (range,13 to 36 months) and 61.5 months (range, 16 to 78 months) in the C-O-BIBR group (P<0.001). Due to differences in follow-up time, with the maturity of surgical techniques, advancements in surgical concepts, and the introduction of patch materials and other biological materials, more patients opted for one-stage reconstruction (n=151, 98.7%) and pre-pectoral bilateral breast reconstruction (54, 35.5%) in R-E-BIBR group, whereas more patients underwent two-stage reconstruction (15, 37.5%) and subpectoral reconstruction (74, 92.5%) in the C-O-BIBR group (P<0.001). In the C-O-BIBR group, all patients (100%) underwent operations in the inpatient ward, while in the R-E-BIBR group, nine patients (11.8%) underwent operations in the day surgery center, and 67 patients (88.2%) in inpatient ward (P=0.026). There were no significant differences in other indicators between the two groups, as shown in **Table 1**.

**Table 1:** Comparison of Demographic and Clinical Characteristics between the Two Groups of Patients with BIBR.

Characteristics	C-O-BIBR group	R-E-BIBR group	P
	N=40, n=80	N=76, n=152	
Mean ± age SD, years	41.2 ± 8.1	41.7 ± 8.8	0.761
Mean BMI ± SD, kg/m^2^	21.6 ± 2.5	21.9 ± 3.1	0.660
Median follow-up time, months	61.5	22	<0.001
Medical treatment status			0.026
Inpatient ward	40 (100)	67 (88.2)	
Day surgery center	0 (0)	9 (11.8)	
Smoking status			0.345
Nonsmoker	39 (97.5)	76 (100)	
Active smoker	1 (2.5)	0 (0)	
Diabetes			0.544
Yes	0 (0)	2 (2.6)	
No	40 (100)	74 (97.4)	
Breast cup size			0.415
≤A	8 (20)	24 (31.6)	
B	24 (60)	39 (51.3)	
≥C	8 (20)	13 (17.1)	
Breast ptosis			0.622
0	31 (77.5)	55 (72.3)	
I	8 (20)	16 (21.1)	
II	1 (2.5)	5 (6.6)	
Histology^#^			0.390
Benign	39 (48.8)	87 (57.2)	
IDC	38 (47.5)	62 (40.8)	
DCIS	3 (3.7)	3 (2.0)	
Indication for mastectomy^#^			0.217
Therapeutic	41 (51.2)	65 (42.8)	
Prophylactic	39 (48.8)	87 (57.2)	
Nipple resection^#^			0.434
No	75 (93.7)	138 (90.8)	
Yes	5 (6.3)	14 (9.2)	
Lymph node surgery^#^			0.492
No surgery	42 (52.5)	90 (59.2)	
SLNB	22 (27.5)	40 (26.3)	
SLNB-ALND/ALND	16 (20.0)	22 (14.5)	
Reconstruction procedure^#^			<0.001
Direct-to-implant	65 (62.5)	151 (98.7)	
Two-stage	15 (37.5)	1 (1.3)	
Reconstruction type^#^			<0.001
Subpectoral	74 (92.5)	82 (54.0)	
Dual-plane	2 (2.5)	16 (10.5)	
Pre-pectoral	4 (5.0)	54 (35.5)	
Pathological T stage^*^(NA=2)			0.831
Tis	6 (16.2)	6 (10.3)	
T1	20 (54.1)	33 (56.9)	
T2	10 (27.0)	18 (31.1)	
Pathological N stage^*^(NA=2)			0.810
N0	26 (70.3)	38 (65.5)	
N1	10 (27.0)	19 (32.8)	
AJCC classification^*^(NA=2)			0.371
0	6 (16.2)	4 (6.9)	
I	18 (48.7)	26 (44.8)	
II	12 (32.4)	27 (46.6)	
ER^*^(NA=4)			0.238
Positive	25 (67.6)	34 (58.6)	
Negative	12 (32.4)	20 (34.5)	
PR^*^(NA=4)			0.254
Positive	23 (62.2)	32 (55.2)	
Negative	14 (37.8)	22 (37.9)	
HER-2^*^(NA=7)			0.087
Positive	11 (67.6)	8 (13.8)	
Negative	25 (29.7)	44 (75.9)	
Neoadjuvant chemotherapy^*^	11 (29.7)	12 (20.7)	0.316
Adjuvant radiation therapy^#^	16 (20.0)	21 (13.8)	0.221
Adjuvant endocrinotherapy^*^	29 (78.4)	36 (62.1)	0.095
Adjuvant chemotherapy^*^	16 (43.2)	35 (60.3)	0.103

C-O-BIBR, conventional open bilateral implant-based breast reconstruction; R-E-BIBR, reverse-sequence endoscopic bilateral implant-based breast reconstruction; BIBR, bilateral implant-based breast reconstruction; SD, standard deviation; BMI, body mass index; IDC, invasive ductal carcinoma; DCIS, ductal carcinoma *in situ*; SLNB, sentinel lymph node biopsy; ALND, axillary lymph node dissection; ER, estrogen receptor; PR, progesterone receptor; NA, not available.

^*^Variable collected for breast cancer patients.

^#^One breast as a unit.

N represents the number of patients.

n represents the number of breasts.

### Cosmetic outcomes and implant-related complications

**Figure 3:**
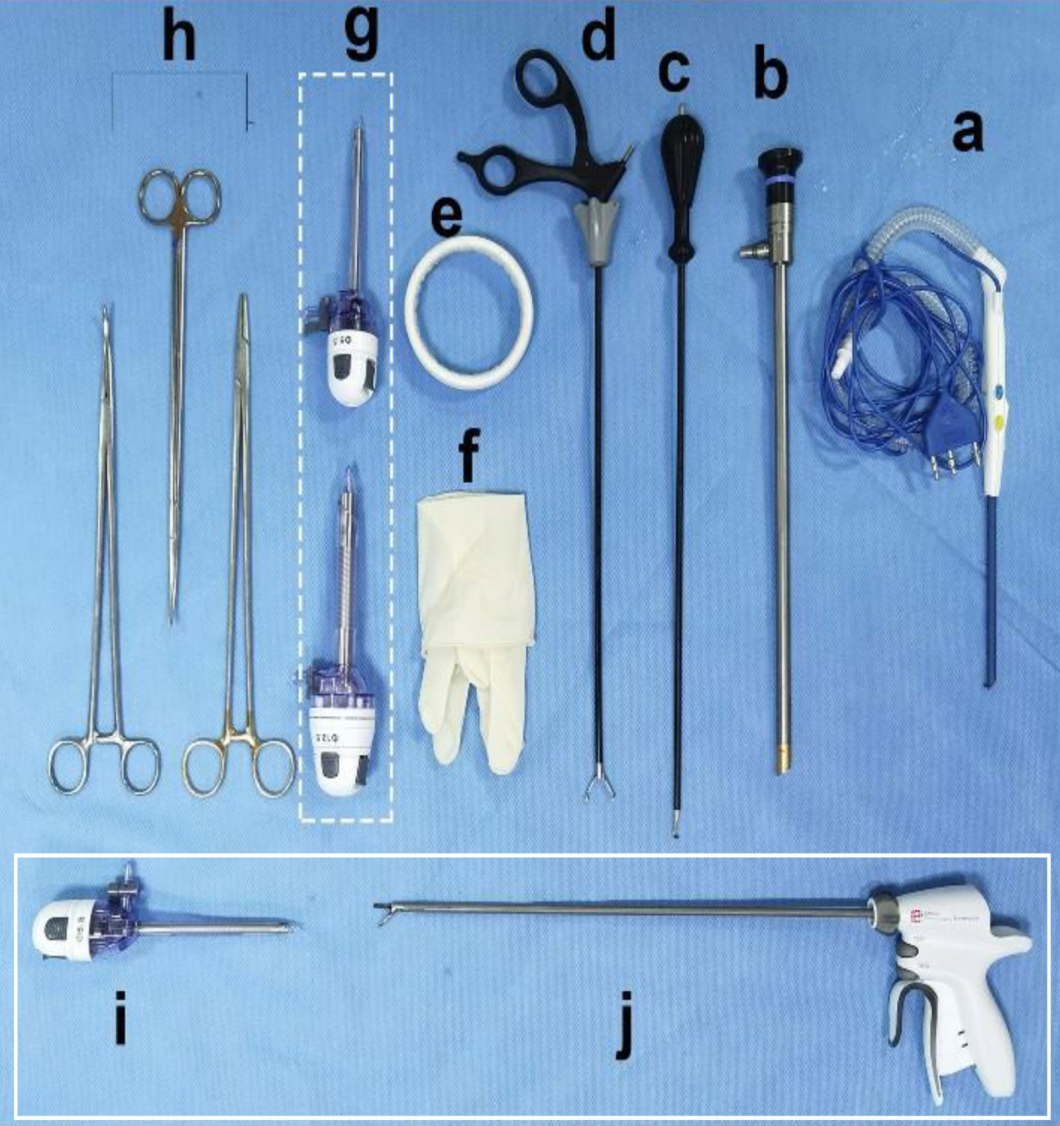
Endoscopic surgical instruments. **(A)** Peng’s multifunctional operative dissector (POMD) (Shuyou Surgical, Hangzhou, China). **(B)** 2D Endoscope cam(30°) (Aesculap Inc, Center Valley, USA). **(C)** Coagulation Hook (Aesculap Inc, Center Valley, USA). **(D)** Grasping Forceps (Aesculap Inc, Center Valley, USA). **(E)** 80-mm Disposable wound protector (Surkon Medical, Wuxi, China). **(F)** Sterile surgical glove (7#) **(G)** + **(I)** Trocars (5.5 mm*2 and 12.5mm, Aesculap Inc, Center Valley, USA). **(H)** Lengthened curved forceps, Lengthened scissor, Lengthened needle holder. Peng’s multifunctional operative dissector (POMD) can be replaced for the common electric scaple; **(I, J)** are standby equipment for beginner, which are not required in most cases).

All patients who received BIBR with tissue expanders had replaced the tissue expanders with prostheses. The mean unilateral breast incision length (12.4 ± 5.3 cm *vs.* 4.6 ± 0.5 cm, P < 0.001), the average incision number [3 (2-4 incisions) *vs.* 2 (all 2 incisions), P < 0.001], and the incision location (P < 0.001) were significantly different between the C-O-BIBR group and the R-E-BIBR group (**Figure 3**). The incision scar was scored by the Scar-Q appearance scale, which was 35.17 ± 9.6 in the C-O-BIBR group and 81.79 ± 12.3 in the R-E-BIBR group (P < 0.001). The patient-reported outcomes (Harris score) and doctor-reported outcomes (Ueda score) show that the excellent rates were 32.5% and 40.0% in the C-O-BIBR group, while 50.0% (P < 0.001) and 75.0% (P < 0.001) in the R-E-BIBR group, respectively. Furthermore, we also found that the incidence of implant-related complications was higher in the C-O-BIBR group [21 (26.2%) *vs.* 21 (13.8%), (P=0.019)] when compared to the R-E-BIBR group (**Table 2**). We divided patients into the satisfied group and the dissatisfied group based on the assessment of both the doctor’s score (Ueda scale) and the patient’s score (Harris scale) were excellent or good, and then the R-E-BIBR procedure was detected as an independent factor to resulted in better cosmetic results through univariate and multivariate logistic regressions, which demonstrates that the aesthetic effect of the R-E-BIBR group is indeed better than that of the C-O-BIBR group (P=0.002) (**Table 3**).

**Table 2:** Cosmetic outcomes, implant-related complications and surgical complications after the Endoscopic Surgery and Traditional Surgery for Bilateral Breast Reconstruction.

	C-O-BIBR group		R-E-BIBR group		P_1_	P_2_
	N=40	n=80	N=76	n=152		
Mean incision length ± SD, cm		12.4 ± 5.3		4.6 ± 0.5		<0.001
Incision location		118		152		<0.001
Inframammary fold incision		22 (18.6)		0 (0)		
Sub-axillary fold incision/other incisions		96 (81.4)		152 (100)		
Incision number						<0.001
2		6 (15.0)		76 (100)		
3		30 (75.0)		0 (0)		
4		4 (10.0)		0 (0)		
Scar-Q	35.17 ± 9.6		81.79 ± 12.3		<0.001	
Harris scale					<0.001	
Excellent	13 (32.5)		44 (58.0)			
Good	13 (32.5)		22 (28.9)			
Fair	12 (30)		9 (11.8)			
Poor	2 (5.0)		1 (1.3)			
Ueda scale					<0.001	
Excellent	20 (50.0)		57 (75.0)			
Good	10 (25.0)		13 (17.1)			
Fair	6 (15.0)		4 (5.3)			
Poor	4 (10.0)		2 (2.6)			
Any implant-related complications		21 (26.2)		21 (13.8)		0.019
Capsular contracture						
III		15 (18.8)		19 (12.5)		
IV		1 (1.3)		0 (0)		
Pectoralis major muscle spasm		3 (3.8)		2 (1.3)		
Implant leakage		1 (1.3)		0 (0)		
Animation deformity		4 (5.0)		2 (1.3)		
Pectoralis major muscle pain		3 (3.8)		0 (0)		
Implant visibility		5 (6.3)		4 (2.6)		
Any surgical complication	25 (62.5)	26 (32.5)	9 (11.8)	10 (6.6)	<0.001	<0.001
Major complication (CDC IIIa-IIIb)	10 (25.0)	11 (13.8)	3 (3.9)	3 (2.0)	0.001	<0.001
Implant loss	6 (15.0)	7 (8.7)	3 (3.9)	3 (2.0)	0.062	0.035
Cellulitis	6 (15.0)	7 (8.7)	3 (3.9)	3 (2.0)	0.062	0.035
Wound dehiscence / flap necrosis	2 (5.0)	2 (2.5)	0 (0)	0 (0)	0.117	0.118
Hematoma	1 (2.5)	1 (1.2)	0 (0)	0 (0)	0.345	0.345
NAC ischemic/ necrotic	1 (2.5)	1 (1.2)	0 (0)	0 (0)	0.345	0.345
Minor complication (CDC I-II)	19 (47.5)	19 (23.8)	6 (7.9)	6 (3.9)	<0.001	<0.001
Surgical site infection	5 (12.5)	5 (6.3)	4 (5.3)	4 (2.6)	0.272	0.281
Wound dehiscence / flap necrosis	4 (10.0)	4 (5.0)	0 (0)	0 (0)	0.013	0.013
Seroma	10 (25.0)	10 (12.5)	2 (2.6)	2 (1.3)	<0.001	0.001
Hematoma	2 (5.0)	2 (2.5)	0 (0)	0 (0)	0.117	0.118

**Table 3:** Univariate and Multivariate Analysis between the satisfactional and dissatisfactional group of breast satisfaction score.

	Univariate analysis		Multivariate analysis	
	OR (95% CI)	P	OR (95% CI)	P
Surgery type		<0.001		0.002
C-O-BIBR	1 [Reference]		1 [Reference]	
R-E-BIBR	10.56 (3.73-29.84)		6.02 (1.90-19.06)	
Age	0.98 (0.93-1.03)	0.585		
BMI	1.11 (0.96-1.29)	0.149		
Breast cup size		0.232		
≤A	1 [Reference]			
B	2.18 (0.66-7.24)	0.200		
≥C	2.80 (0.68-11.49)	0.153		
Breast ptosis		0.873		
0	1 [Reference]			
I	1.25 (0.43-3.63)	0.670		
II	0.75 (0.08-6.88)	0.804		
Reconstruction type		0.071		0.382
Subpectoral	1 [Reference]			
Dual-plane	10.91 (1.40-84.96)	0.022		
Pre-pectoral	7.00 (0.36-135.51)	0.198		
Indication for mastectomy		0.155		
Therapeutic	1 [Reference]			
Prophylactic	0.33 (0.07-1.52)			
Lymph node surgery		0.163		
No surgery	1 [Reference]			
SLNB	0.53 (0.09-3.14)	0.493		
SLNB-ALND/ALND	1.66 (0.27-10.03)	0.577		
Implants type		<0.001		0.017
Tissue expander	1 [Reference]		1 [Reference]	
Direct-to-implant	13.51 (4.07-44.80)		5.02 (1.32-18.99)	
Nipple resection	0.96 (0.24-3.76)	0.956		
Radiation therapy	0.26 (0.10-0.65)	0.004		0.099
Chemotherapy	0.81 (0.33-1.97)	0.647		
Endocrinotherapy	0.71 (0.28-1.79)	0.477		
Neoadjuvant chemotherapy	0.54 (0.19-1.53)	0.252		

### Comparison of surgical complications

Compared with the C-O-BIBR group, the R-E-BIBR group is associated with a lower incidence of any surgical complications, counted by the number of breasts (32.5% and 6.6%, P<0.001). The most common major complication was implant loss in both the C-O-BIBR group and the R-E-BIBR group (8.7% and 2.0%, P <0.001). In terms of minor complications, the most common complication in the C-O-BIBR group was seroma, which was significantly different from that in the R-E-BIBR group (12.5% and 1.3%, P=0.001), while in the R-E-BIBR group was surgical site infection, whose incidence rate was still has a lower tendency than that in the C-O-BIBR group (1.3% and 2.6%, P=0.281). In addition, there were still differences in wound dehiscence/flap necrosis between the two groups (5.0% and 0%, P=0.013) (**Table 2**). Furthermore, due to baseline data mismatch, we conducted univariate and multivariate logistic analysis on baseline data, and still found that the rate of surgical complications in the R-E-BIBR group was lower than that in the C-O-BIBR group, with the difference being statistically significant (**Table 4**). And we also found that the incidence of complications was higher in IMF incision compared with sub-axillary fold incision (P=0.007) (**Table 5**).

**Table 4:** Univariate and Multivariate Analysis of Any and major Complications after Bilateral Breast Reconstruction.

	Any surgical complication				Major surgical complication			
	Univariate analysis		Multivariate analysis		Univariate analysis		Multivariate analysis	
	OR (95% CI)	P	OR (95% CI)	P	OR (95% CI)	P	OR (95% CI)	P
Age	0.98 (0.94-1.03)	0.529			1.01 (0.94-1.07)	0.877		
BMI	1.09 (0.97-1.24)	0.136			0.95 (0.78-1.17)	0.589		
Breast cup size		0.865				0.228		
≤A	1 [Reference]				1 [Reference]			
B	1.18 (0.48-2.86)	0.720			5.84 (0.71-47.90)	0.100		
≥C	1.35 (0.45-4.01)	0.593			3.26 (0.27-38.48)	0.348		
Breast ptosis		0.819				0.824		
0	1 [Reference]				1 [Reference]			
I	0.96 (0.39-2.37)	0.981			0.69 (0.14-3.39)	0.649		
II	0.51 (0.06-4.12)	0.528			1.52 (0.16-14.36)	0.715		
Surgery type		<0.001		0.001		0.002		0.002
C-O-BIBR group	1 [Reference]		1 [Reference]		1 [Reference]		1 [Reference]	
R-E-BIBR group	0.16 (0.07-0.36)		0.13 (0.04-0.42)		0.13 (0.03-0.47)		0.12 (0.03-0.48)	
Reconstruction type		0.040		0.058		0.675		
Subpectoral	1 [Reference]				1 [Reference]			
Dual-plane	13.46 (1.79-101.16)	0.012			5.31 (0.87-8.08)	0.997		
Pre-pectoral	14.25 (1.36-149.01)	0.027			4.46 (0.64-6.89)	0.385		
Indication for mastectomy		<0.001		0.998		0.006		0.006
Therapeutic	1 [Reference]				1 [Reference]		1 [Reference]	
Prophylactic	0.17 (0.01-0.13)				0.06 (0.01-0.44)		0.06 (0.01-0.44)	
Lymph node surgery		<0.001		0.353		0.009		0.250
No surgery	1 [Reference]				1 [Reference]			
SLNB	18.75 (4.13-84.99)	<0.001			12.81 (1.51-108.84)	0.019		
SLNB-ALND/ALND	56.54 (12.26-260.77)	<0.001			26.73 (3.18-224.92)	0.003		
Implants type		<0.001		0.053		0.003		0.295
Tissue expander	1 [Reference]				1 [Reference]			
Direct-to-implant	0.12 (0.05-0.28)				0.18 (0.06-0.56)			
Nipple resection	3.04 (1.08-8.68)	0.037		0.444	1.75 (0.21-14.62)	0.604		
Radiation therapy	10.56 (4.66-24.13)	<0.001		0.542	3.21 (1.02-10.26)	0.047		0.961
Chemotherapy	1.16 (0.56-2.40)	0.694			1.05 (0.35-3.13)	0.931		
Endocrinotherapy	0.52 (0.24-1.15)	0.105			0.72 (0.24-2.12)	0.546		
Neoadjuvant chemotherapy	0.34 (0.16-0.76)	0.008		0.572	0.60 (0.18-1.99)	0.402		

BMI, body mass index; SLNB, sentinel lymph node biopsy; ALND, axillary lymph node dissection; C-O-BIBR, conventional open bilateral implant-based breast reconstruction; R-E-BIBR, reverse-sequence endoscopic bilateral implant-based breast reconstruction.

**Table 5:** Comparation between inframammary fold incision and sub-axillary fold incision.

	Inframammary fold incision	Sub-axillary fold incision	P
Any complication			0.007
Yes	6 (27.3)	10 (6.6)	
No	16 (72.7)	142 (93.4)	
Scar-Q	40.18 ± 10.3	80.95 ± 12.9	<0.001
Ueda scale			0.066
Excellent	6 (54.5)	57 (75.0)	
Good	0 (0)	13 (17.1)	
Fair	2 (18.2)	4 (5.3)	
Poor	3 (27.3)	2 (2.6)	
Harris scale			0.343
Excellent	6 (54.5)	44 (58.0)	
Good	2 (18.2)	22 (28.9)	
Fair	0 (0)	9 (11.8)	
Poor	3 (27.3)	1 (1.3)	

### Tumor safety

**Figure 4:**
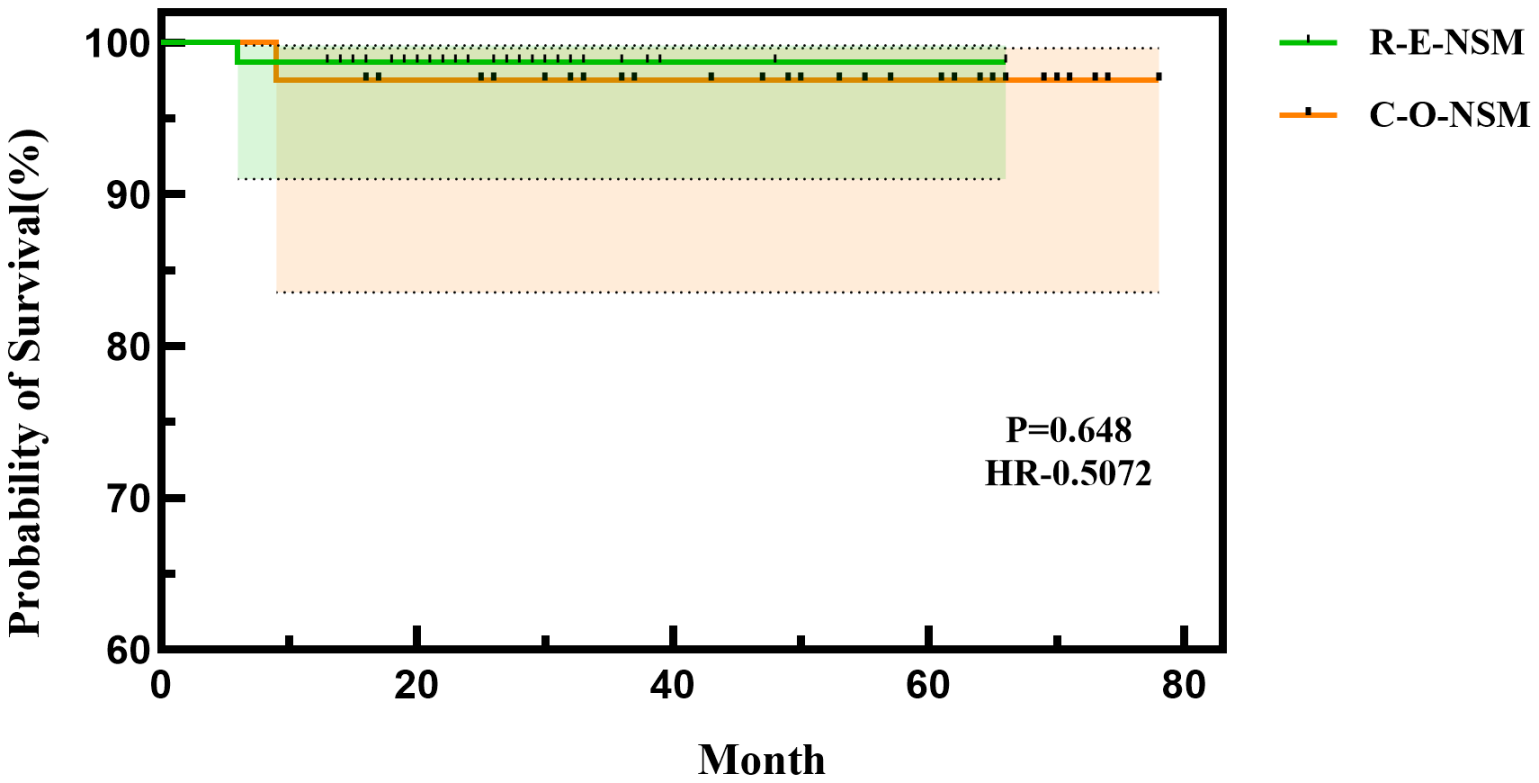
Comparison of survival curves between the two groups.

In the C-O-BIBR group, one patient developed bone metastasis 14 months after surgery, which was treated by endocrine therapy. In the R-E-BIBR group, one patient suffered local recurrence in the chest, axilla, and supraclavicular 6 months after surgery and was treated by re-surgery. The 24 months of disease-free survival of the R-E-BIBR group and the C-O-BIBR group were 98.7% and 97.5%, respectively (P = 0.648) (**Figure 4**).

### Operation time and changes in surgery types

**Figure 5:**
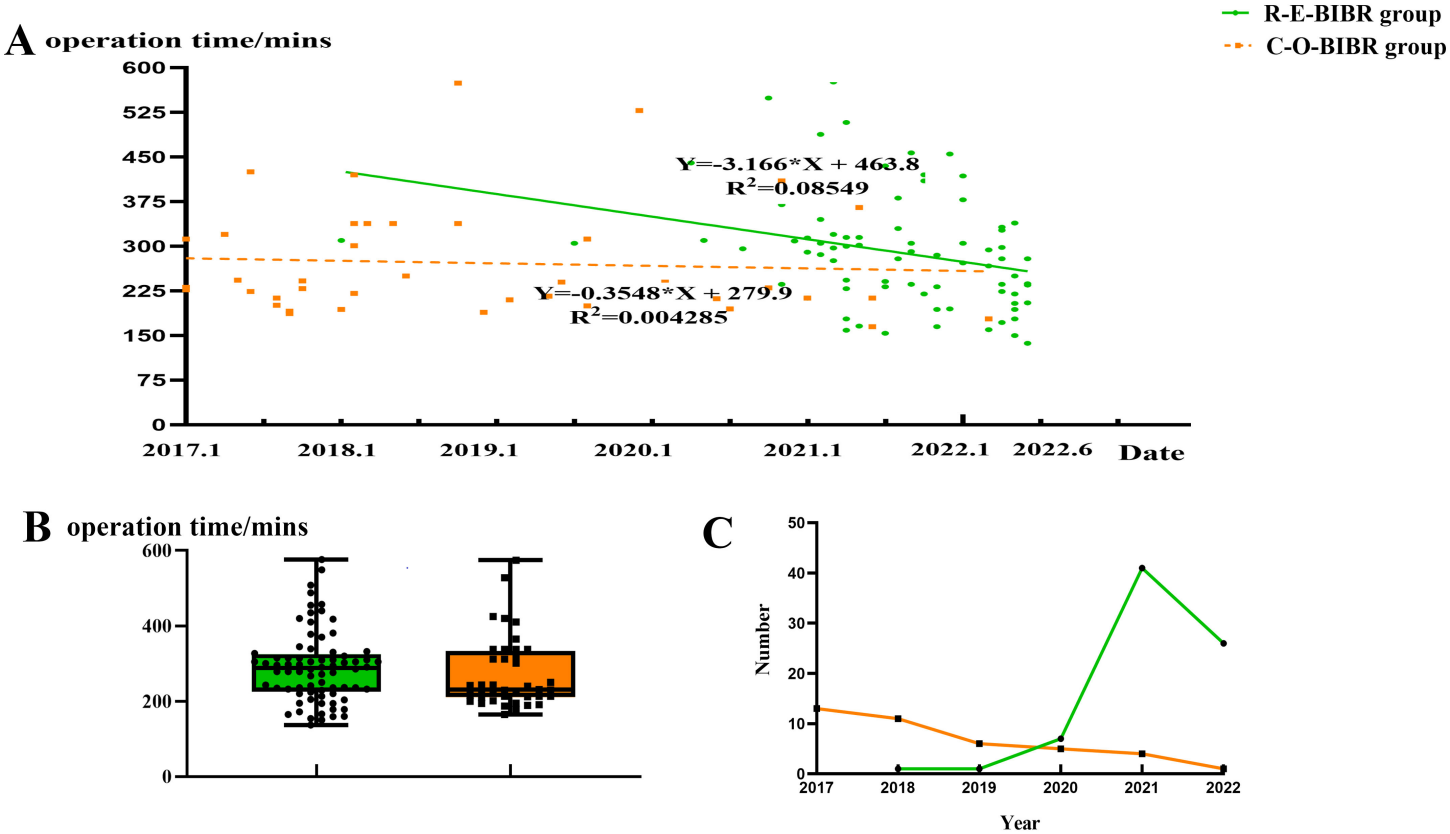
**(A)** Scatter plot of linear regression analysis of operation time between the two groups. **(B)** Line chart of surgery types changing with year. **(C)** Box scatter diagram of operation time in two groups.

The mean operative time of the C-O-BIBR group was 271.9 ± 95.3 mins, and that of the R-E-BIBR group was 290.2 ± 95.2 mins, which was not significantly different (P = 0.327). From the regression curve, we found that the operation time decreased in both groups, but the decreasing trend was more pronounced in the endoscopic group (P = 0.010) (**Figures 5A, B**). Over time, endoscopic surgeries gradually surpassed conventional open procedures in number (**Figure 5C**).

## Discussion

Along with the increase in bilateral breast reconstruction rate, surgeons pay greater attention to the advantages and drawbacks of operation methods (3). As conventional endoscopic surgery is difficult to perform and time-consuming, it has not become the standard of unilateral breast reconstruction, let alone endoscopy- or robot-assisted BIBR (20). However, in this study, we introduced a new technique (R-E-NSM), by comparing the conventional open and the new endoscopic methods for BIBR in the same center in terms of operation time, postoperative complications, and cosmetic effects, we found that surgical complications were significantly reduced and cosmetic results were significantly improved in the R-E-BIBR group (23–25). At the same time, R-E-NSM with BIBR did not significantly increase the operation time.

With the pursuit of quality of life and the premise of ensuring tumor safety, many patients choose breast reconstruction after mastectomy, not only considering the survival result but also weighing the cosmetic effect. Some studies have reported that endoscopic or robotic surgeries can hide the incision under the armpit, avoid noticeable breast scars, improve postoperative beauty, and effectively reduce the incidence of prosthesis loss caused by incision dehiscence and prosthesis exposure (2, 36). Cosmetic improvement also has been widely regarded as an advantage of endoscopic and robotic surgery. The number of incisions can also affect the effectiveness of cosmetic studies. Endoscopic surgeries often use axillary incisions, while open surgeries may involve 3 or 4 incisions, with 3 incisions commonly seen in unilateral cancer contralateral benign lesions or prophylactic resections, and 4 incisions commonly seen in bilateral cancers.

Despite the clear cosmetic benefits of endoscopic or robotic surgery, it is baffling that this technique has not been widely adopted over the past three decades. Operation time and the need for special equipment directly affect the promotion of a surgical technique. When compared to conventional open surgery, it is a fact that both conventional endoscopic and robotic surgery require more equipment and significantly longer operation times because breasts are parenchymal organs that require adequate working space to exposure and enough tension during resection at the same time. It is reported that the average reconstruction time of the robot-assisted surgery is 322 min, while the previously reported average reconstruction time of endoscopy-assisted surgery is 347 min (20). The reason for the long operation time is that they applied the traditional sequential method in the past. If somebody used special retractors or suspension devices to help expose the surgical resection site, the uneven force distribution and limited visibility would result in complicated procedures and a higher risk of surgical complications. If somebody used the CO_2_ inflation method, it would provide better exposure to the surgical field. However, certain tissues like glands or pectoralis major muscles during sub-pectoral reconstruction can be challenging to expose. This is because suspension cannot be easily achieved using retractors when the axillary incision is closed by an incision protective sleeve. These two situations perfectly illustrate the idiom that you can’t have your cake and eat it. Thus, nearly all breast surgeons believe that endoscopic or robotic surgeries are almost impossible and not suitable for promotion.

However, due to the use of the innovative reverse-sequence method and “HUAXI Hole 1”, the surgeons can skillfully adopt the pressure of the CO_2_ and the gravity of the tissue to carry out layered dissection, easily remove the whole glands, and obviously shorten the operation time (23). The idea of the reverse-sequence method makes the gas (CO_2_) not only play the effect of clear vision but also play the role of universal retractors, and the force is uniform, so it can significantly improve the efficiency of the operation, which achieves the purpose of having your cake and eat it. Compared with conventional liposuction endoscopy-assisted and robot-assisted nipple-sparing mastectomy, the operation time is significantly shortened (25). After overcoming the initial learning curve, our innovative endoscopic technique can reduce bilateral endoscopic NSM with IBR operation time to 137 min (25). In our study, the mean operation time of the R-E-BIBR group was just 20 minutes longer than that of the C-O-BIBR group but was not statistically significant. After analyzing carefully, we can even find that the operation time of unilateral endoscopic breast reconstruction may be less than that of unilateral open reconstruction, for we know that conventional bilateral surgery is often performed on both sides by two surgeons at the same time while endoscopic surgery must be completed on one side and then the other side. Therefore, whether the operation time of the endoscopic group is really longer than that of the conventional open group remains to be discussed. Of course, more patients who underwent endoscopic BIBR in this study opted for pre-pectoral reconstruction, which is another factor affecting the operation time. Based on advancements in surgical concepts and techniques, as well as considering the individual breast conditions of patients, the choice between pre-pectoral and subpectoral reconstruction is made. Initially, subpectoral reconstruction was predominantly chosen. However, in later stages, pre-pectoral reconstruction was preferred for patients with thicker and more robust skin flaps. About the selection of surgery type, it is based on patient’s acceptance and understanding of the procedure, safety considerations, and aesthetic outcomes, decided through shared discussions. There is no bias in the selection of surgical approaches.

The significant reduction in the operation time and no of special surgical equipment needed for R-E-NSM with BIBR indicate that it can be promoted. So, we need to pay attention to its surgical safety. Previous studies on the incidence of complications after breast reconstruction vary widely due to the different definitions, with the overall incidence ranging from 5.8% to 52% (8, 21). In this study, the incidence of surgical complications in the R-E-BIBR group was much lower than that in the C-O-BIBR group. Analyzing the reasons, we think that there are the following: firstly, in endoscopic surgery, incision dehiscence does not occur because there is no incision on the breast envelope; secondly, endoscopic magnification enhances visualization, allowing for more detailed anatomical assessment, leading to preservation of subcutaneous vessels and reduced risk of avascular necrosis of the skin; thirdly, endoscopic surgery makes axillary crease incision, which is far from the NAC, while conventional open surgery is mainly radial incision, close to the nipple and areola (4). Therefore, the risk of NAC ischemia after traditional surgery was higher than that in the endoscopic group; fourthly, endoscopic surgery is “no touch” surgery, leading to a lower incidence of postoperative infection and prosthesis loss (37).

There was 1 patient who experienced local recurrence 6 months after surgery in the endoscopic group and 1 patient who experienced metastasis 14 months after surgery in the conventional open group, whose 24 months’ disease-free survival time was 98.7% and 97.5%, respectively (P=0.648). There is no difference in 24 months disease-free survival time between the two groups, which proved again the safety of this method.

This study found that there are benefits of performing bilateral endoscopic reconstruction with implants, which can reduce postoperative surgery-related complications and improve cosmetic outcomes. However, our study also has limitations. First, it is a retrospective study, and there is selection bias. Second, our sample size was small. Third, the follow-up period was a little short. Therefore, the conclusions drawn from this study need to be further confirmed by prospective studies.

In conclusion, the innovative R-E-NSM with IBR makes up for the long operation time of previous endoscopic surgeries and has significant advantages in reducing complication rates and improving the cosmetic results of the postoperative breasts.

Ethical approval: All procedures performed in studies involving human participants were in accordance with the ethical standards of the institutional and/or national research committee and with the 1964 Helsinki Declaration and its later amendments or comparable ethical standards. And for this type of study formal consent is not required.

## Data Availability

The raw data supporting the conclusions of this article will be made available by the authors, without undue reservation.
